# An improved IPT-PLL technology for single-phase grid-connected inverters in complex power grid conditions

**DOI:** 10.1038/s41598-024-62702-y

**Published:** 2024-05-28

**Authors:** Zhang Tao, Dong Dezhi

**Affiliations:** 1 Research Center for Photovoltaic System Engineering of Ministry of Education, Hefei University of Technology, Hefei, 230009 China; 2 Dongguan Jishi Electric Co., Ltd, Dongguan, 523808 China; 3 College of Electrical Engineering, Tongling College, Tongling, 244000 China

**Keywords:** Phase-locked loop (PLL), Complex power grid, Frequency adaptivity, Harmonic interference, Resonant controller, Engineering, Electrical and electronic engineering

## Abstract

Aiming at the common problems of frequency variations and harmonics in complex power grids, an improved inverse Park transform phase locked loop (IPT-PLL) technology for single-phase converters suitable for micro grid systems is proposed. Firstly, in the phase detection of PLL, the α component of Park transformation is selected as the reference voltage, and its orthogonal component is constructed using a 1/4 fundamental period delay method. Secondly, Lagrange interpolation polynomials are introduced to approximate fractional delay to solve the problem of delay calculation errors caused by frequency changes. Thirdly, in order to compensate for the poor ability of traditional proportional integral (PI) regulators, multi resonant controllers are superimposed to suppress low order harmonic disturbances. Finally, the design method and system performance of the PI regulator and each resonant controller are analyzed theoretically. The experimental results show that the proposed improved IPT-PLL method has strong adaptability to complex power grids. It can significantly improve the tracking performance of power grid frequency, suppress the interference of low order harmonics and DC bias. And it has good dynamic and static performance.

## Introduction

**Figure 1:**
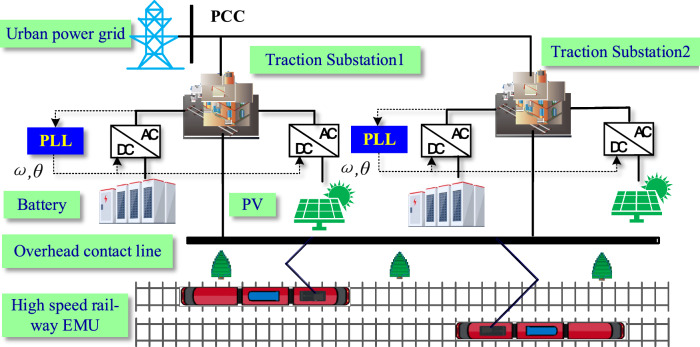
Single phase microgrid of traction power supply.

With the rise of electrified transportation, the energy consumption in this sector has become a pressing issue. The interplay between transportation, energy, and the environment has gained significant attention. Consequently, the fusion of renewable energy sources and low-carbon transportation has emerged as a key area of focus within the realm of electrical engineering. Microgrids, constructed from renewable energy such as photovoltaic and wind power generation and energy storage are favored in the field of electrical transportation^1,2^. Figure 1 illustrates a microgrid power supply system designed for railway electrification locomotives and electric multiple units (EMU). Each traction electrical substation, in addition to being connected to the AC power grid, is equipped with photovoltaic panels, energy storage units, and other components of specific capacities. The DC/AC grid-connected inverter (GCI) serves as the intermediary link between the photovoltaic systems, energy storage, and the AC power grid. To ensure the safe and efficient operation of microgrids, various technical challenges need to be addressed, with grid synchronization technology being a crucial aspect^3,4^. However, the traction substation load consists of the traction converter for high-speed trains, the power supply for home signal, and railway shifter, all of which demonstrate strong nonlinearity. The harmonic currents produced by these devices travel through the impedance of the traction power grid line, leading to background harmonics in the voltage of the traction substation transformer secondary side. Moreover, the substantial and rapid changes in traction power in EMUs can easily cause fluctuations in power supply voltage frequency. The voltage in the traction power grid often exhibits complex characteristics such as harmonics, amplitude variations, DC offset, frequency fluctuations, which significantly impact the performance of synchronization algorithms and may even result in damage to the GCIs^5,6^. So accurately determining the frequency, phase, and amplitude information of the fundamental component of the voltage in the complex grid is essential for designing the control system of the GCI. This information is crucial for ensuring the safe and stable operation of the traction power supply system^7^.

Phase lock loops (PLL) is a widely synchronization method used in GCIs. It mainly includes three parts which are phase detector (PD), loop filter (LF) and voltage control oscillator (VCO). Single-phase PLL can be divided into two types: the stationary coordinate system PLL and the synchronous reference frame PLL (SRF-PLL) according to different PDs.

For the stationary coordinate system PLL, sine signal multiplication is often used to achieve phase detection, which inevitably suffers from interference of twice the frequency and affects the control accuracy of PLL^8^. In order to solve the problem of double frequency oscillation,^9^ and^10^ introduced high-order low-pass filters and notch filters after PD, respectively, to improve the steady-state performance of PLL by setting a lower filter bandwidth, but its dynamic performance will be greatly affected.

The SRF-PLL borrows from the three-phase voltage PLL principle and utilizes rotating coordinate transformation to realize phase detection. This method has gained widespread application due to its fast dynamic response and ease of software implementation^11,12^. However, for single-phase AC microgrid systems, there is only one voltage vector and rotational coordinate transformation cannot be directly applied as in the three-phase SRF-PLL. Therefore, different methods have been devised to design a Quadrature Signal Generator (QSG), which are then used for PD through rotational coordinate transformation. Commonly used QSG methods include the Second Order Generalized Integrator (SOGI)^13,14^ and T/4 (where T is the fundamental period) delay^15^. Various advanced SOGI methods have been developed to address DC offset in PLL, including additional offset estimation loops, cascade SOGI, modified SOGI, complex coefficient filters, in-loop dq-frame DSC, notch filters, and moving average filter-based SOGI-PLL^16^. In^17^, an additional integral branch was added to the front-end SOGI to suppress DC offset, a sliding average filter was used in the back-end PLL loop instead of the integral controller of the PLL. While these enhanced SOGI-PLL algorithms effectively tackle the impact of DC offset on PLL performance, they may not account for frequency variations and harmonics in complex grid voltage. For the T/4 delay method, in^18^, an adaptive delay SPF-PLL algorithm was constructed based on the deviation between the estimated frequency and the nominal frequency of the power grid. However, it did not consider issues such as the DC offset and harmonics of the grid voltage. In^19^, the T/4 delay + Inverse Park Transformation (IPT) method was used to construct orthogonal components, effectively suppressing the DC offset of the grid voltage. However, this method did not consider the accuracy of T/4 digital delay implementation when the grid frequency changes, which will affect the output accuracy of the PLL. In^20^ a multi-harmonic decoupling compensation network was established by performing Park transformation and its inverse transformation at different harmonic frequencies, the influence of grid harmonics on the PLL is eliminated. Due to the need for a large number of Park transforms, the calculation speed of PLL is affected. A high-pass filter was used to extract the harmonic components in the dq components after Park transformation in^21^, and then the difference between the extracted components and the original dq components was taken to filter out the grid voltage harmonics. But the adverse effects caused by the phase lag of the high-pass filter on the harmonic filtering algorithm were not addressed.

In addition, methods such as Discrete Fourier Transform (DFT)^22^, Hilbert Transform^23^, Kalman Filtering^24^, and Double Complex Coefficient Filtering^25^ have also been used for voltage signal orthogonal component construction. However, these methods have various issues, such as requiring a large amount of controller memory or experiencing significant phase locking errors and low-frequency oscillations in complex power grid environments.

This paper introduces an improved PLL technology building upon the conventional IPT-PLL to address synchronization control challenges of the GCI in complex grids with DC offset, voltage harmonics and frequency variations. Firstly, the α component of the IPT-PLL reverse Park transformation is used as the reference and the orthogonal components is constructed by the use of the T/4 delay method. Secondly, the Lagrange interpolation polynomial is used to estimate the fractional delay, which improves the accuracy of the T/4 delay algorithm and enhances the frequency adaptability of the PLL. Thirdly, a PI + multi-resonant controller is used to construct the LF method, which helps to mitigate the impact of voltage harmonics in the complex power grid. Design methods of the PI regulator and multi resonant controllers of LF are analyzed. At last, good performances of the proposed improvement scheme are discussed.

## Basic principles of IPT-PLL

### Principle of PD in IPT-PLL

**Figure 2:**
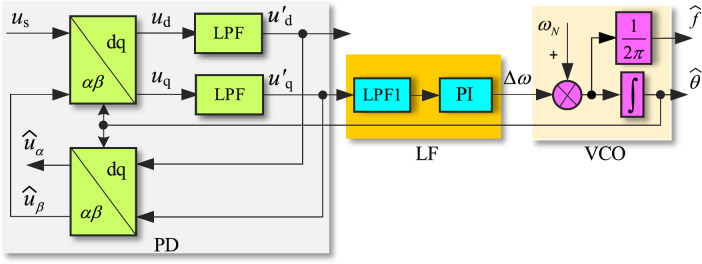
Conventional IPT-PLL.

IPT-PLL structure is shown in Fig. 2^18^, where PD consists of three parts: Park transformation, low pass filter (LPF), and inverse Park transformation. The grid voltage *u*_s_ and the output signal $\widehat{u}_{\beta }$ of inverse Park transformation are transformed into dq components *u*_d_ and *u*_q_ through Park transformation. And the high-order harmonics of dq components are filtered out by LPF to obtain the DC form $u_{{\text{d}}}{\prime}$ and $u_{{\text{q}}}{\prime}$, which are then transformed into $\widehat{u}_{\alpha }$ and $\widehat{u}_{\beta }$ through inverse Park transformation. The q-axis component $u_{{\text{q}}}{\prime}$ from LPF is taken as the output of PD, the error angular frequency $\Delta \omega$ is generated through the LPF and PI regulator of LF, and then it is summed with the nominal angular frequency *ω*_N_ (*ω*_N_ = 100π rad/s) of the complex power grid’s fundamental voltage. And the PLL output $\widehat{\theta }$ and the power grid frequency observation signal $\widehat{f}$ are obtained from VCO.

According to the PD shown in Fig. 2, the transfer function *F*(*s*) of the first-order LPF is defined as

1 $$F{(}s{)} = \frac{{\omega_{c} }}{{s + \omega_{c} }}$$

where *ω*_c_ represents the cutoff frequency of the LPF.

The time-domain relationship between the input of the Park transform in Fig. 2 and the output of the inverse Park transform can be derived as

2 $$\left[ {\begin{array}{*{20}c} {\widehat{u}_{\alpha } {(}t{)}} \\ {\widehat{u}_{\beta } {(}t{)}} \\ \end{array} } \right] = \left[ {\begin{array}{*{20}c} {{\text{cos}}\widehat{\theta }} & { - {\text{sin}}\widehat{\theta }} \\ {{\text{sin}}\widehat{\theta }} & {{\text{cos}}\widehat{\theta }} \\ \end{array} } \right] \times \left\{ {\left[ {\begin{array}{*{20}c} {f(t)} & 0 \\ 0 & {f(t)} \\ \end{array} } \right] * \left( {\left[ {\begin{array}{*{20}c} {{\text{cos}}\widehat{\theta }} & {{\text{sin}}\widehat{\theta }} \\ { - {\text{sin}}\widehat{\theta }} & {{\text{cos}}\widehat{\theta }} \\ \end{array} } \right] \times \left[ {\begin{array}{*{20}c} {u_{s} {(}t{)}} \\ {\widehat{u}_{\beta } {(}t{)}} \\ \end{array} } \right]} \right)} \right\}$$

where * represents the convolution operator, *f*(*t*) is the unit impulse response of the LPF。

In terms of Euler's formula, Park transformation matrix can be expressed as

3 $$\left[ {\begin{array}{*{20}c} {{\text{cos}}\widehat{\theta }} & {{\text{sin}}\widehat{\theta }} \\ { - {\text{sin}}\widehat{\theta }} & {{\text{cos}}\widehat{\theta }} \\ \end{array} } \right] = \frac{1}{2}\left[ {\begin{array}{*{20}c} {e^{{j\widehat{\omega }t}} + e^{{ - j\widehat{\omega }t}} } & {je^{{j\widehat{\omega }t}} - je^{{ - j\widehat{\omega }t}} } \\ { - je^{{j\widehat{\omega }t}} + je^{{ - j\widehat{\omega }t}} } & {e^{{j\widehat{\omega }t}} + e^{{ - j\widehat{\omega }t}} } \\ \end{array} } \right]$$

where $$\widehat{\theta } = \widehat{\omega }t$$.

According to Eq. (3) and the property of frequency shift, the Laplace transform of Eq. (2) can be obtained as

4 $$\left[ {\begin{array}{*{20}c} {\widehat{u}_{\alpha } {(}s{)}} \\ {\widehat{u}_{\beta } {(}s{)}} \\ \end{array} } \right] = \frac{1}{2}\left[ {\begin{array}{*{20}c} {F{(}s + j\widehat{\omega }{)} + H{(}s - j\widehat{\omega }{)}} & { - jF{(}s + j\widehat{\omega }{)} + jH{(}s - j\widehat{\omega }{)}} \\ {jF{(}s + j\widehat{\omega }{)} - jH{(}s - j\widehat{\omega }{)}} & {F{(}s + j\widehat{\omega }{)} + H{(}s - j\widehat{\omega }{)}} \\ \end{array} } \right] \cdot \left[ {\begin{array}{*{20}c} {u_{s} {(}s{)}} \\ {\widehat{u}_{\beta } {(}s{)}} \\ \end{array} } \right]$$

The transfer functions ${{\widehat{u}_{\alpha } {(}s{)}} \mathord{\left/ {\vphantom {{\widehat{u}_{\alpha } {(}s{)}} {u_{s} {(}s{)}}}} \right. \kern-0pt} {u_{s} {(}s{)}}}$ and ${{\widehat{u}_{\beta } {(}s{)}} \mathord{\left/ {\vphantom {{\widehat{u}_{\beta } {(}s{)}} {u_{s} {(}s{)}}}} \right. \kern-0pt} {u_{s} {(}s{)}}}$ derived by (4) can be expressed as (5) and (6) respectively.

5 $$T_{\alpha } {(}s{)} = \frac{{\widehat{u}_{\alpha } {(}s{)}}}{{u_{s} {(}s{)}}} = \frac{{\omega_{c} s}}{{s^{2} + \omega_{c} s + \widehat{\omega }^{2} }}$$

6 $$T_{\beta } {(}s{)} = \frac{{\widehat{u}_{\beta } {(}s{)}}}{{u_{s} {(}s{)}}} = \frac{{\omega_{c} \widehat{\omega }}}{{s^{2} + \omega_{c} s + \widehat{\omega }^{2} }}$$

**Figure 3:**
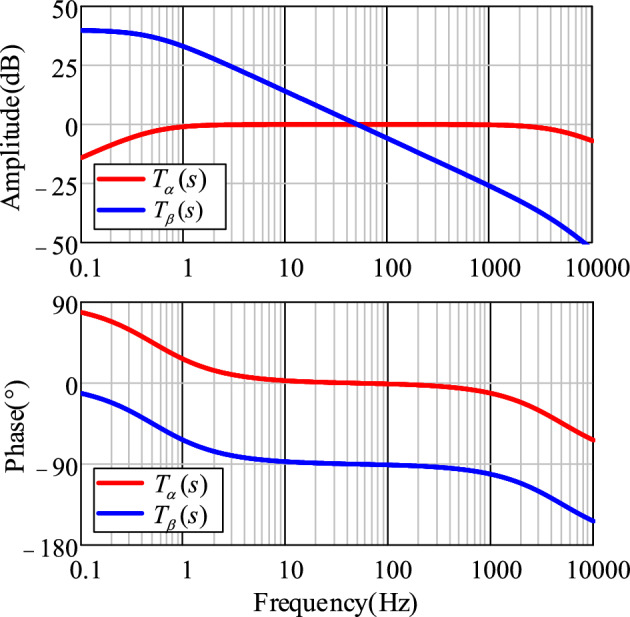
Bode plot of IPT transfer function.

The Bode plots of Eqs. (5) and (6) are shown in Fig. 3, where *ω*_c_ = 2000π rad/s. It can be seen that *T*_α_(*s*) is a second-order band-pass filter with angular frequency centered on $\widehat{\omega }$, which can filter DC and high-frequency components of the complex power grid voltage. *T*_*β*_ (*s*) is a second-order LPF with an amplitude of 1 at a frequency of 50 Hz and a phase shift of − 90°. It can be inferred that $\widehat{u}_{\beta } {(}s{)}$ represents the quadrature component of the grid voltage *u*_s_.

### Some problems with IPT-PLL

For a single-phase complex power grid, it generally includes a DC offset, fundamental and harmonic voltage harmonics. The complex grid voltage *u*_s_(*t*) can be written as

7 $$u_{s} {(}t{)} = U_{0} + U{\text{cos(}}\omega t + \varphi {)} + \sum\limits_{h = 3,5,7...} {U_{h} {\text{cos(}}h\omega t + \varphi_{h} {)}}$$

where *U*_0_, *U*, and *U*_h_ represent the amplitudes of the DC component, fundamental component, and *h*th harmonic component of the grid voltage respectively. *ω* is the angular frequency of the fundamental voltage, while *φ* and *φ*_h_ represent the phases of the fundamental and *h*th harmonic voltages, respectively. If PLL output angular frequency $\widehat{\omega } = \omega$, the steady-state components of $\widehat{u}_{\alpha } {(}t{)}$ and $\widehat{u}_{\beta } {(}t{)}$ are shown in (8) and (9) can be calculated from Eqs. (5)–(7).

8 $$\widehat{u}_{\alpha } {(}t{)} = U{\text{cos(}}\omega t + \phi {)} + \frac{1}{2}\sum\limits_{h = 3,5,7...} {\left[ {\left| {T_{\alpha } {(} - jh\omega {)}} \right| + \left| {T_{\alpha } {(}jh\omega {)}} \right|} \right]U_{h} {\text{cos}}} \left[ {h\omega t + \phi_{h} + \angle T_{\alpha } {(}jh\omega {)}} \right]$$

9 $$\widehat{u}_{\beta } {(}t{)} = U_{0} + U{\text{sin(}}\omega t + \phi {)} + \frac{1}{2}\sum\limits_{h = 3,5,7...} {\left[ {\left| {T_{\beta } {(} - jh\omega {)}} \right| + \left| {T_{\beta } {(}jh\omega {)}} \right|} \right]U_{h} {\text{sin}}} \left[ {h\omega t + \phi_{h} + \angle T_{\beta } {(}jh\omega {)}} \right]$$

where $\left| {T_{\alpha } {(}jh\omega {)}} \right|$、$\left| {T_{\alpha } {(} - jh\omega {)}} \right|$、$\left| {T_{\beta } {(}jh\omega {)}} \right|$ and $\left| {T_{\beta } {(} - jh\omega {)}} \right|$ are moduli of $T_{\alpha } {(}s{)}$ and $T_{\beta } {(}s{)}$, $\angle T_{\alpha } {(}jh\omega {)}$ and $\angle T_{\beta } {(}jh\omega {)}$ are phase angles of $T_{\alpha } {(}s{)}$ and $T_{\beta } {(}s{)}$ when $s = \pm jh\omega$ respectively.

By substituting (8) and (9) into the Park transformation matrix, the expression for *u*_q_ can be obtained as

10 $$u_{q} {(}t{)} = U{\text{sin(}}\theta - \widehat{\theta }{)} + U_{0} {\text{cos}}\widehat{\theta } + \sum\limits_{h = 3,5,7...} {n{[(}h + 1{)}\omega t,{(}h - 1{)}\omega t{]}}$$

where

$$\begin{gathered} n{[(}h + 1{)}\omega t,{(}h - 1{)}\omega t{]} = \frac{{\left[ {\left| {T_{\alpha } ( - jh\omega )} \right| + \left| {T_{\alpha } (jh\omega )} \right|} \right]U_{h} }}{4}\left\{ {{\text{sin[(}}h + 1{)}\omega t + \phi_{h} + \angle T_{\alpha } {(}jh\omega {)]} - {\text{sin[(}}h - 1{)}\omega t + \phi_{h} + \angle T_{\alpha } {(}jh\omega {)]}} \right\} \hfill \\ + \frac{{\left[ {\left| {T_{\beta } {(} - jh\omega {)}} \right| + \left| {T_{\beta } {(}jh\omega {)}} \right|} \right]U_{h} }}{4}\left\{ {{\text{sin[(}}h + 1{)}\omega t + \phi_{h} + \angle T_{\beta } {(}jh\omega {)]} - {\text{sin[(}}h - 1{)}\omega t + \phi_{h} + \angle T_{\beta } {(}jh\omega {)]}} \right\} \hfill \\ \end{gathered}$$

**Figure 4:**
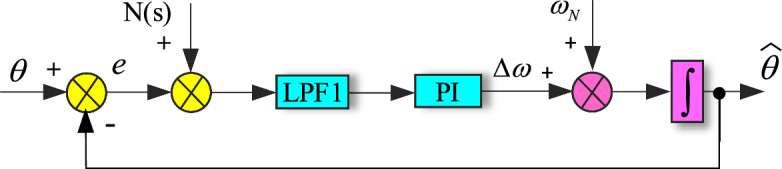
Linear model of conventional IPT-PLL.

According to Eq. (10), in steady state, ${\text{sin(}}\theta - \widehat{\theta }{)}$ is approach to $\theta - \widehat{\theta }$. The DC component of the grid voltage can be expressed as $U_{0} {\text{cos}}\widehat{\theta }$, which is an AC component with a frequency of $\widehat{\omega }$. The *h*th voltage harmonic is transformed into (*h* ± 1)th harmonic component. Consider the DC and harmonic components of the grid voltage as disturbances *N*(s), which is the frequency domain expression of *n*[(*h* + 1)*ω*t,(*h *− 1)*ω*t] to the PLL. The linear model block diagram of IPT-PLL is shown in Fig. 4.

The transfer function of the PI controller and LPF1 of LF in Fig. 4 are given as (11) and (12) respectively

11 $$W_{{{\text{PI}}}} {(}s{)} = k_{p} + \frac{{k_{i} }}{s}$$

where *k*_p_ and *k*_i_ are the proportional and integral coefficients of the PI controller.

12 $$W_{{{\text{LPF1}}}} {(}s{)} = \frac{{\omega_{c1} }}{{s + \omega_{c1} }}$$

where *ω*_c1_ is the cutoff frequency of the LPF in LF of the PLL.

According to Fig. 4, it can be seen that the closed-loop transfer function under the input signal *θ*(*s*) and the disturbance signal *N*(*s*) is the same. It can be given by

13 $$G_{c} {(}s{)} = \frac{{{(}k_{p} s + k_{i} {)}\omega_{{{\text{c1}}}} k_{vco} }}{{s^{3} + \omega_{{{\text{c1}}}} s^{2} + k_{p} \omega_{{{\text{c1}}}} s + k_{i} \omega_{{{\text{c1}}}} k_{vco} }}$$

where *k*_*vco*_ is the gain of VCO, and *k*_*vco*_ = *U*.

Through some simple theoretical derivations, the output $\widehat{\theta }{(}s{)}$ and the error *E*(*s*) in Fig. 4 can be obtained as

14 $$\left\{ \begin{gathered} \widehat{\theta }{(}s{)} = G_{c} {(}s{)[}N{(}s{)} + \theta {(}s{)]} \hfill \\ E{(}s{)} = \frac{{{(}s^{3} + \omega_{{{\text{c1}}}} s^{2} {)}\theta {(}s{)}}}{{s^{3} + \omega_{{{\text{c1}}}} s^{2} + k_{p} \omega_{{{\text{c1}}}} s + k_{i} \omega_{{{\text{c1}}}} \omega_{{{\text{c1}}}} }} - \frac{{{(}k_{p} s + k_{i} {)}\omega_{{{\text{c1}}}} k_{vco} N{(}s{)}}}{{s^{3} + \omega_{{{\text{c1}}}} s^{2} + k_{p} \omega_{{{\text{c1}}}} s + k_{i} \omega_{{{\text{c1}}}} k_{vco} }} \hfill \\ \end{gathered} \right.$$

From (14), it can be seen that the closed-loop phase error *E*(*s*) based on IPT contains the low-frequency component of *N*(s) is shown in Fig. 13. This will cause phase fluctuation of PLL output, which in turn affecting the stable operation of the GCI. In addition, the conventional IPT-PLL has not solved the issue of phase-locked loop output double frequency ripple caused by grid frequency variations.

## Improved IPT-PLL

**Figure 5:**
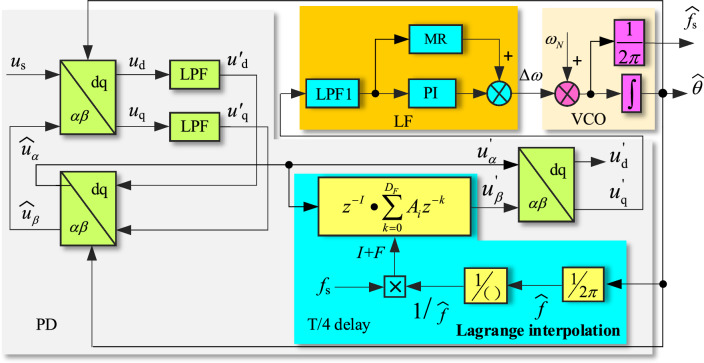
Improved IPT-PLL block diagram.

This paper proposes an improved strategy for the shortcomings of the conventional IPT-PLL, as shown in Fig. 5. Since *T*_α_(*s*) is a bandpass filter, it can filter out the DC component and higher-order harmonics of the grid voltage, while preserving all the information of the fundamental frequency. In this paper, $\widehat{u}_{\alpha } {(}s{)}$ is taken as the research object, its delay T/4 is used to obtain the corresponding orthogonal component $u_{\beta }{\prime} {(}s{)}$. And the fractional delay caused by grid frequency variations is estimated using Lagrange interpolation polynomials to solve the problem of computational accuracy. Then the q-axis component is obtained through the Park transformation, and PD is realized. Considering the influence of low-order harmonics in the grid voltage on the PLL, a multi-resonant (MR) controller is paralleled with the PI controller to improve the anti-interference performance of PLL against low order harmonics.

### Improved T/4 Delay Realization

The transfer function of the T/4 delay element can be represented as:

15 $$D{(}z{)} = z^{{ - {{f_{s} } \mathord{\left/ {\vphantom {{f_{s} } {{(}4 \cdot \hat{f}{)}}}} \right. \kern-0pt} {{(}4 \cdot \hat{f}{)}}}}} = z^{{ - {(}I + F{)}}}$$

where *f*_s_ is the switching frequency of the GCI, $\widehat{f}$ is the output frequency of the PLL. If the grid frequency varies, $f_{s} /\widehat{f}$ may not be an integer. It can be represented as the sum of its integer part *I*, and its fractional part *F*.

In order to improve the accuracy of T/4 delay calculation, the fractional order delay element can be approximated using Lagrange interpolation^26^, $z^{ - F}$ can be approximated by

16 $$z^{ - F} \approx \sum\limits_{k = 0}^{N} {d_{k} z^{ - k} }$$

where N is the highest degree of the Lagrange interpolation polynomial, $$d_{k} { = }\prod\limits_{\begin{subarray}{l} i = 0 \\ i \ne k \end{subarray} }^{N} {\frac{F - i}{{k - i}}} \begin{array}{*{20}c} , & {{(}k = 0,1,2...N{)}} \\ \end{array}$$ are polynomial coefficients.

Table 1 shows the calculation formulas for the corresponding interpolation polynomial coefficients as *N* varies from 1 to 4.

**Table 1:** Lagrange interpolation polynomial coefficients for fractional-order delay elements. (N = 1, 2, 3, 4).

N	*d*(0)	*d*(1)	*d*(2)	*d*(3)	*d*(4)
N = 1	– 1– F	F			
N = 2	(F– 1)(F– 2)/2	– F(F– 2)	F(F– 1)/2		
N = 3	– (F– 1)(F– 2)(F– 3)/6	F (F– 2)(F– 3)/2	– F(F– 1) (F– 3)/2	F(F– 1)(F– 2)/6	
N = 4	(F– 1)(F– 2)(F– 3)(F– 4)/24	– F(F– 2)(F– 3)(F– 4)/6	F(F– 1)(F– 3)(F– 4)/4	– F(F– 1)(F– 2)(F– 4)/6	F(F– 1)(F– 2)(F– 3)/24

**Figure 6:**
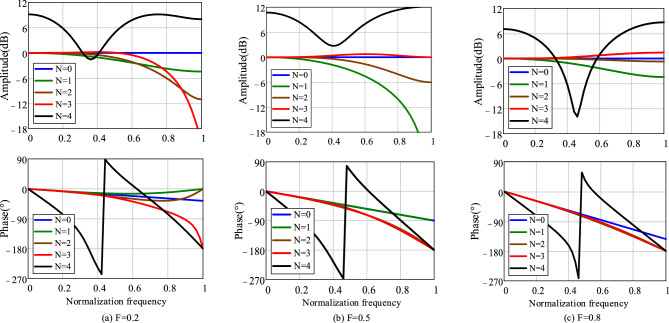
Approximation of different fractional-order delay elements with different Nth-order interpolation polynomials.

Figure 6 shows the frequency response curves of different-order Lagrange interpolation polynomials approximating the fractional-order delay element at *F* = 0.2, 0.5, and 0.8. Here, *N* = 0 represents the original fractional-order delay element. It can be observed that when N is set to 1 ~ 3, the calculated interpolation polynomials can approximate the original delay element within a certain frequency range. However, when *N* is set to 4 or higher, the obtained interpolation polynomials exhibit significant differences in both magnitude and phase compared to the original element. Taking into account the magnitude-frequency and phase-frequency characteristics, polynomial with *N* = 3 is chosen to approximate the fractional-order delay element.

**Figure 7:**
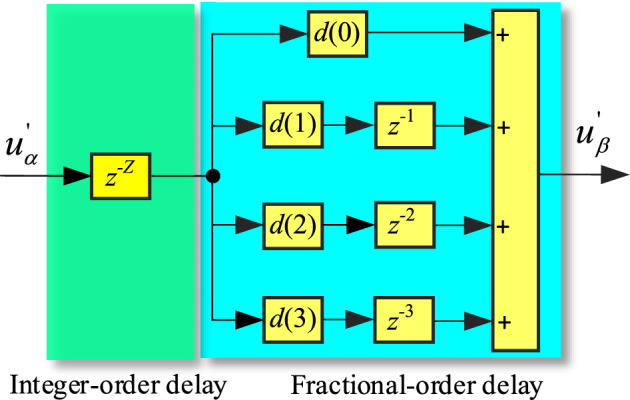
Implementation method of T/4 delay with frequency adaptability.

Based on the previous analysis, the implementation method of T/4 delay with frequency adaptivity can be obtained as shown in Fig. 7.

### Harmonic issues and solutions in IPT-PLL

#### Harmonic issues in IPT-PLL

By using the improved phase estimation method for IPT proposed in this paper, the complex power grid voltage expression (7) and the bandpass filter characteristics of *T*_*α*_(*s*) can be used to obtain the result shown in Fig. 5.

By using the improved IPT phase detection method proposed in this paper, the expression of $u_{\alpha }{\prime}$, as shown in Fig. 5, can be derived from the complex power grid voltage expression (7) and the bandpass filter characteristics of *T*_α_(s).

17 $$u_{\alpha }{\prime} = U{\text{cos(}}\omega t + \phi {)} + \sum\limits_{h = 3,5,7...} {U_{h}{\prime} {\text{cos(}}h\omega t + \phi_{h} {)}}$$

where $U_{h}{\prime}$ represents the amplitude of the harmonic voltage contained in $u_{\alpha }{\prime}$, which has a certain attenuation relative to $U_{h}$ due to the effect of filter *T*_α_(s).

Under the effect of T/4 delay, the orthogonal signal $u_{\beta }{\prime}$ corresponding to $u_{\alpha }{\prime}$ can be written as

18 $$u_{\beta }{\prime} = U{\text{cos[}}\omega {(}t - \frac{T}{4}{)} + \phi {]} + \sum\limits_{h = 3,5,7...} {U_{h}{\prime} {\text{cos[}}h\omega {(}t - \frac{T}{4}{)} + \phi_{h} {]}}$$

After some trigonometric transformations, Eqs. (17) and (18) can be rewritten as

19 $$\left[ {\begin{array}{*{20}c} {u_{\alpha }{\prime} } \\ {u_{\beta }{\prime} } \\ \end{array} } \right] = \left[ {\begin{array}{*{20}c} {U{\text{cos(}}\omega t + \phi {)}} \\ {U{\text{sin(}}\omega t + \phi {)}} \\ \end{array} } \right] + \left[ {\begin{array}{*{20}c} {\sum\limits_{h = 3,7,11...} {U_{h}{\prime} {\text{cos(}} - h\omega t - \phi_{h} {)}} } \\ {\sum\limits_{h = 3,7,11...} {U_{h}{\prime} {\text{sin(}} - h\omega t - \phi_{h} {)}} } \\ \end{array} } \right] + \left[ {\begin{array}{*{20}c} {\sum\limits_{h = 5,9,13...} {U_{h}{\prime} {\text{cos(}}h\omega t + \phi_{h} {)}} } \\ {\sum\limits_{h = 5,9,13...} {U_{h}{\prime} {\text{sin(}}h\omega t + \phi_{h} {)}} } \\ \end{array} } \right]$$

From Eq. (19), it can be seen that $u_{\alpha }{\prime}$ and $u_{\beta }{\prime}$ constructed by the T/4 delay module include fundamental components, negative sequence harmonic components such as the 3rd, 7th, and 11th harmonics, as well as positive sequence harmonic components such as the 5th, 9th, and 13th harmonics. By performing a Park transformation on $u_{\alpha }{\prime}$ and $u_{\beta }{\prime}$, the q-axis component $u_{{\text{q}}}^{^{\prime\prime}}$ is given by

20 $$u_{q}^{^{\prime\prime}} {(}t{)} = U{\text{sin(}}\theta - \widehat{\theta }{)} + \sum\limits_{{{\text{n}} = 3,7,11...}} {U_{h}{\prime} {\text{sin[(}} - h - 1{)}} \omega t - \phi_{h} {]} + \sum\limits_{h = 5,9,13...} {U_{h}{\prime} {\text{sin[(}}h - 1{)}\omega t + \phi_{h} {]}}$$

According to Eq. (20), it can be seen that the q-axis component based on the improved IPT phase detection contains harmonic components such as ± 4, ± 8, and so on, which have an impact on the PLL.

#### The solution to the harmonic problem

The harmonic components of $u_{{\text{q}}}^{^{\prime\prime}}$ can be regarded as disturbance signals to the PLL. Insufficient gain of the PI controller at harmonic frequencies prevents the elimination of low-order harmonic signals on the PLL. The resonant controller features a sine transfer function with a specified resonant frequency, where the gain can theoretically reach infinity. This effectively reduces steady-state error to zero in response to step changes in a reference signal at that frequency^27,28^. This paper proposes implementing parallel resonant controller of various harmonic frequencies alongside a conventional PLL PI controller to suppress harmonic interference signals.

**Figure 8:**
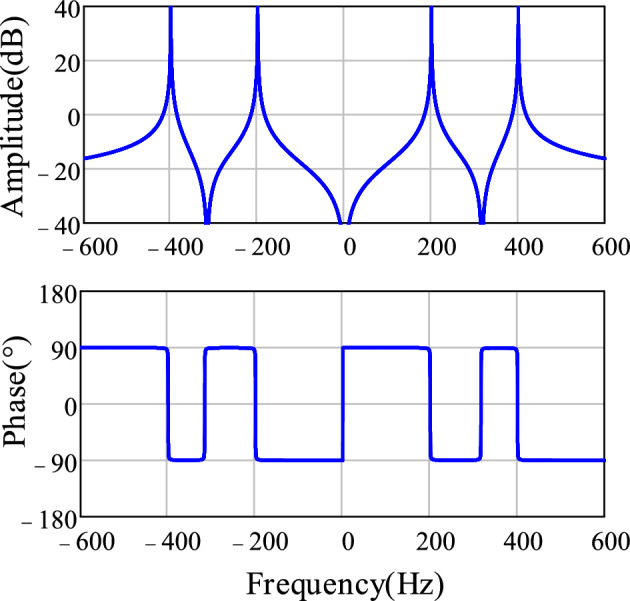
Bode plot of the MR controller.

The transfer function of the MR controller is given as follows

21 $$G_{{{\text{MR}}}} {(}s{)} = \sum\limits_{h = 4,8...} {\frac{{2k_{rh} \omega_{ch} s}}{{s^{2} + 2\omega_{ch} s + \left( {h\hat{\omega }} \right)^{2} }}}$$

where $k_{rh}$ and $\omega_{ch}$ are the resonant gain and cutoff frequency of the resonant controller at *h*th harmonic frequencies, respectively. Considering the suppression effect of bandpass filter *T*_α_(s) on high-order harmonics of the grid voltage, it can be assumed that the orthogonal components $u_{\beta }{\prime}$ contain harmonics from the 3^rd^ to the 9th order. Therefore, resonant controllers at the 4th and 8th harmonic frequencies can be added to the PI controller in the LF of the PLL. Figure 8 shows the Bode plot of the MR controller (*h* = 4, 8) with *k*_*rh*_ = 50 and *ω*_*ch*_ = 4 rad/s. From the figure, it can be seen that the resonant controller at the *h*th harmonic frequency has infinite gain at the ± *h*th harmonic frequencies, which enables the suppression of disturbance signals at the ± *h*th harmonic frequencies.

## Tuning procedure of the improved IPT-PLL

In the improved IPT-PLL shown in Fig. 5, assuming the system is in steady state, the grid voltage contains low-order harmonics such as the 3rd to 9th harmonics. Equation (20) can be approximated as follows

22 $$u_{q}^{^{\prime\prime}} {(}t{)} \approx U{(}\theta - \widehat{\theta }{)} + \sum\limits_{{{\text{n}} = 3,7}} {U_{h}{\prime} {\text{sin[(}} - h - 1{)}} \omega t - \phi_{h} {]} + \sum\limits_{h = 5,9} {U_{h}{\prime} {\text{sin[(}}h - 1{)}\omega t + \phi_{h} {]}}$$

**Figure 9:**
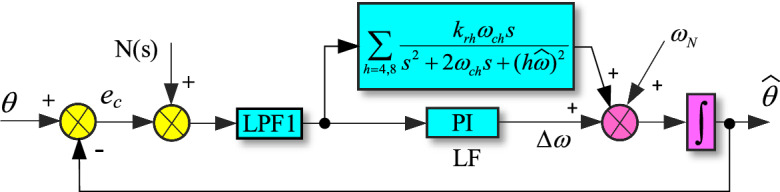
Linear model of improved IPT-PLL.

The improved IPT-PLL linear model structure diagram can be obtained as shown in Fig. 9.

The corresponding open-loop transfer function *G*_o_(*s*) is

23 $$G_{{\text{o}}} {(}s{)} = \left[ {k_{p} + \frac{{k_{i} }}{s} + \sum\limits_{h = 4,8} {\frac{{2k_{rh} \omega_{ch} s}}{{s^{2} + 2\omega_{ch} s + \left( {h\hat{\omega }} \right)^{2} }}} } \right]\frac{{U\omega_{c1} }}{{s{(}s + \omega_{c1} {)}}}$$

According to the conclusion obtained from^26^, the PI controller determines the bandwidth and DC gain of the open-loop system, while the resonant controller determines the gain at the resonant frequency of the open-loop system. It can be considered that the PI controller and the resonant controller are decoupled, and the phase margin (PM) of the control system can be used as a constraint to separately design each controller.

### Design of PI controller

The open-loop transfer function *G*_o1_(*s*) of the IPT-PLL linear model, as shown in Fig. 9, can be obtained by ignoring the influence of the MR controller.

24 $$G_{{{\text{o1}}}} {(}s) = \frac{{K{(}1 + s/\omega_{z} {)}}}{{s^{2} {(}1 + s/\omega_{p} {)/}\omega_{z} }}$$

where $$\omega_{z} = {{k_{i} } \mathord{\left/ {\vphantom {{k_{i} } {k_{p} }}} \right. \kern-0pt} {k_{p} }}$$, $$\omega_{p} = \omega_{c1}$$, $$K = k_{p} U$$。

The crossover frequency *ω*_*co*_, where the open loop gain is unity, can be solved by $\left| {G_{{{\text{o}}1}} {(}j\omega_{co} {)}} \right| = 1$.

25 $$\omega_{co} = \frac{{K \cdot {\text{cos(}}\phi_{p} {)}}}{{{\text{sin(}}\phi_{z} {)}}}$$

where $$\phi_{z} = {\text{arctan(}}{{\omega_{co} } \mathord{\left/ {\vphantom {{\omega_{co} } {\omega_{z} }}} \right. \kern-0pt} {\omega_{z} }}{)}$$, $$\phi_{p} = {\text{arctan(}}{{\omega_{co} } \mathord{\left/ {\vphantom {{\omega_{co} } {\omega_{p} }}} \right. \kern-0pt} {\omega_{p} }}{)}$$.

The PM expressed in (26) of the open-loop system can be obtained from Eq. (24).

26 $${\text{PM}} = {\text{arctan(}}{{\omega_{co} } \mathord{\left/ {\vphantom {{\omega_{co} } {\omega_{z} }}} \right. \kern-0pt} {\omega_{z} }}{)} - {\text{arctan(}}{{\omega_{co} } \mathord{\left/ {\vphantom {{\omega_{co} } {\omega_{p} }}} \right. \kern-0pt} {\omega_{p} }}{)}$$

Differentiating Eq. (26) with respect to *ω*_*co*_ and setting it to zero. Then *ω*_*co*_ can be calculated as

27 $$\omega_{co} = \sqrt {\omega_{p} \cdot \omega_{z} } = \sqrt {\frac{{k_{i} \omega_{c1} }}{{k_{p} }}}$$

Hence the phase margin (PM) of the PLL control loop is maximized, if the control system loop bandwidth *ω*_*co*_ satisfies Eq. (27). In the meantime, ${\text{sin(}}\phi_{z} {\text{) = cos(}}\phi_{p} {)}$, $\omega_{co} = K$.

Define the parameter $b = {{\omega_{p} } \mathord{\left/ {\vphantom {{\omega_{p} } {\omega_{z} }}} \right. \kern-0pt} {\omega_{z} }}$, and PM can be calculated as

28 $${\text{PM}} = {\text{arctan}}\frac{b - 1}{{2\sqrt b }}$$

It can be seen that the maximum PM is exclusively determined by b.

**Figure 10:**
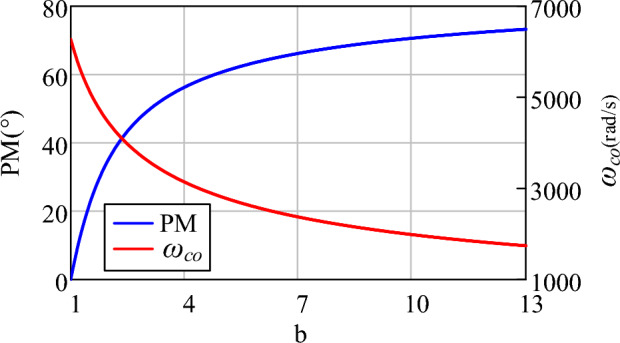
Phase margin and crossover frequency with variation for different b values.

In this paper, the value of *ω*_c1_ for the LPF in LF is set to 2000π rad/s. According to (27) and (28), Fig. 10 illustrates the PM and *ω*_*co*_ for different b values from 1 to 13. It shows that, if the PM is set to 50°, the value of b can be chosen as 3 and *ω*_*co*_ = 3626 rad/s which are derived by (28) and (27) respectively. From (25) and (27), the parameters of PI controller can be computed as *k*_*p*_ = 11.7, *k*_*i*_ = 24,495.

### Design of MR

The MR controller shown in Fig. 9 includes two sets of parameters: resonant gain and open-loop cutoff frequency *ω*_ch_. Considering frequency selectivity^29^, it is possible to choose the same open-loop cutoff frequency for each resonance controller. In this paper, *ω*_ch_ is set to 4 rad/s. And then the resonant gains *k*_r4_ and *k*_r8_, which are 4 times and 8 times the fundamental frequency resonant controllers respectively, are calculated based on the phase constraints. Taking the example of a PI controller with a resonant controller that is 4 times the fundamental frequency ($4\widehat{\omega }$), the range of values for *k*_r4_ is discussed in this paper. The open-loop transfer function* G*_02_(s) of IPT-PLL with PI and a resonant controller under $4\widehat{\omega }$ is written as

29 $$G_{02} {(}s{)} = \left[ {k_{p} + \frac{{k_{i} }}{s} + \frac{{2k_{r4} \omega_{c4} s}}{{s^{2} + 2\omega_{c4} s + \left( {4\hat{\omega }} \right)^{2} }}} \right]\frac{{U\omega_{c1} }}{{s{(}s + \omega_{c1} {)}}}$$

In order to ensure system stability, the phase of system at $4\widehat{\omega }$ must satisfy the following inequality

30 $$180^{{\text{o}}} + \left\{ {\left. {{\text{arg[}}G_{o2} {(}s{)]}} \right|_{{s = j4\widehat{\omega }}} } \right\} \times \frac{{180^{{\text{o}}} }}{\pi } > 0^{{\text{o}}}$$

**Figure 11:**
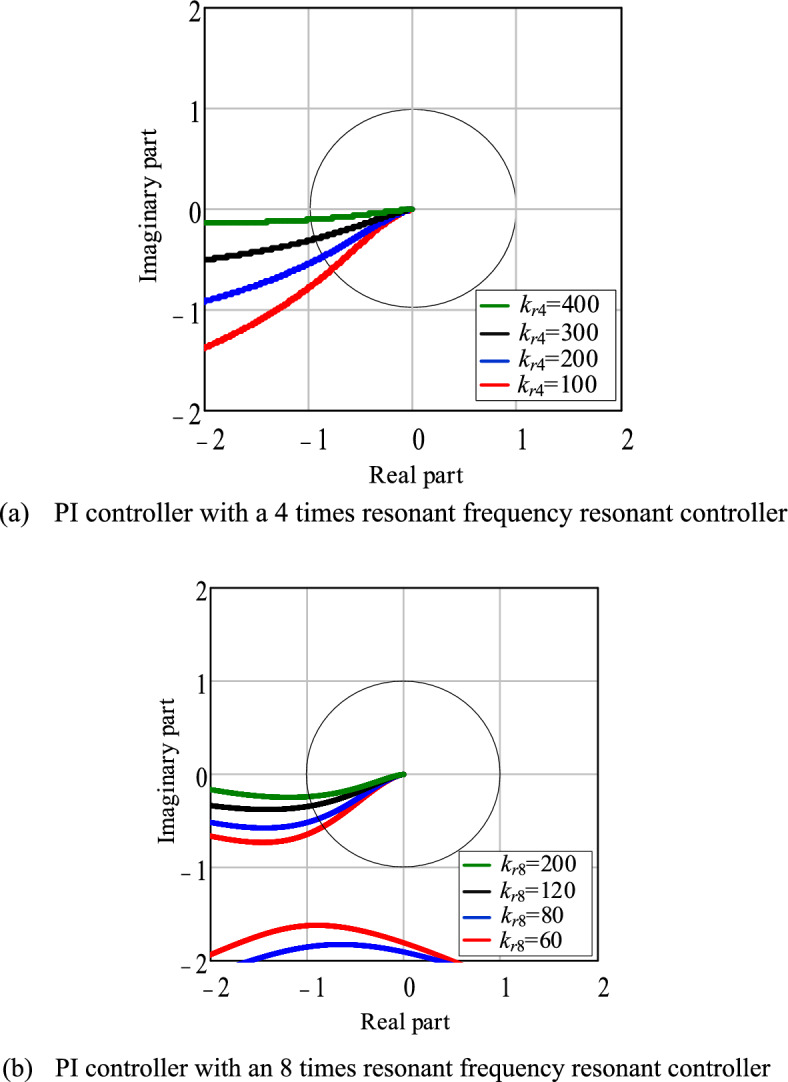
PLL Nyquist diagram corresponding to different resonant controller gains.

Figure 11a shows the Nyquist plot of *G*_02_(*s*) for different values of *k*_r4_. It can be observed that as *k*_r4_ increases, the phase at the resonant frequency approaches − 180°. For *k*_r4_ < 400, Eq. (30) holds true. In this paper, *k*_r4_ is chosen to be 300. And the phase at the resonant frequency is − 160°, providing a phase margin of 20° at the resonant frequency. Similarly, the open-loop transfer function *G*_03_(*s*) of the PI controller with an 8 times resonant frequency resonant controller of IPT-PLL is given by

31 $$G_{03} {(}s{)} = \left[ {k_{p} + \frac{{k_{i} }}{s} + \frac{{2k_{r8} \omega_{c8} s}}{{s^{2} + 2\omega_{c8} s + \left( {8\hat{\omega }} \right)^{2} }}} \right]\frac{{U\omega_{c1} }}{{s{(}s + \omega_{c1} {)}}}$$

According to the Nyquist plot shown in Fig. 11b, when* k*_r8_ is set to 200, the phase at the resonant frequency is − 165°, and the open-loop system has a phase margin of 15° at the resonant frequency.

**Figure 12:**
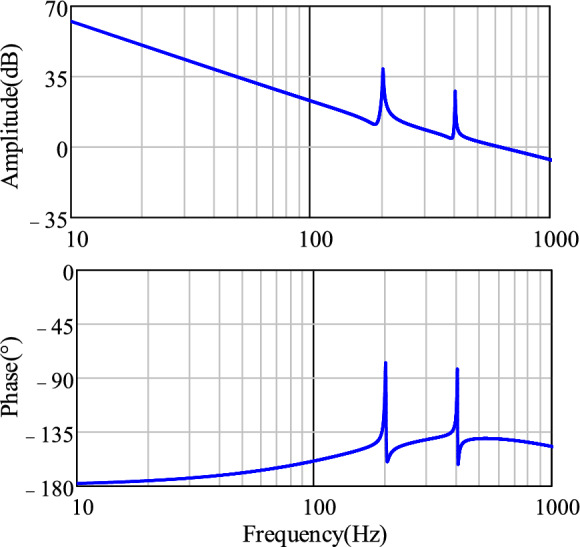
Bode plot of the proposed improved PLL open-loop transfer function.

The Bode plot of the open-loop transfer function (23) for the improved IPT-PLL scheme, utilizing the determined parameters of the PI controller and MR controller, is depicted in Fig. 12. It is clear that the addition of the resonant controller does not impact the stability of the initial system with the PI controller, as each resonant controller operates independently. At specific resonant frequencies, there is a significant gain enhancement, effectively reducing harmonic interference at those frequencies within the PLL control loop.

### Performance Analysis of the Improved IPT-PLL

For the open-loop transfer function (23), the frequency domain expression of the error *E*_c_(s) in the improved IPT-PLL scheme shown in Fig. 9 can be obtained as

32 $$E_{{\text{c}}} {(}s{)} = \frac{{\theta {(}s{)}}}{{1 + G_{o} {(}s{)}}} - \frac{{G_{o} {(}s{)}N{(}s{)}}}{{1 + G_{o} {(}s{)}}}$$

**Figure 13:**
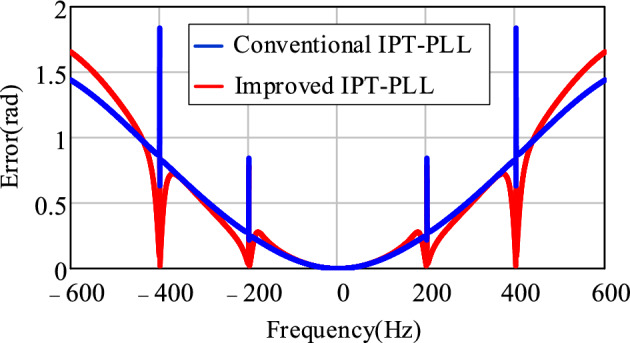
Comparison curve of PLL control loop error signal.

Comparing the expression of the error signal *E*(*s*) for the conventional IPT-PLL in (13), the amplitude-frequency characteristics of the PLL error signal for both schemes are plotted in Fig. 13. It can be observed that the improved IPT-PLL effectively suppresses the influence of low-order harmonics from the grid on the PLL.

## Experimental results

**Figure 14:**
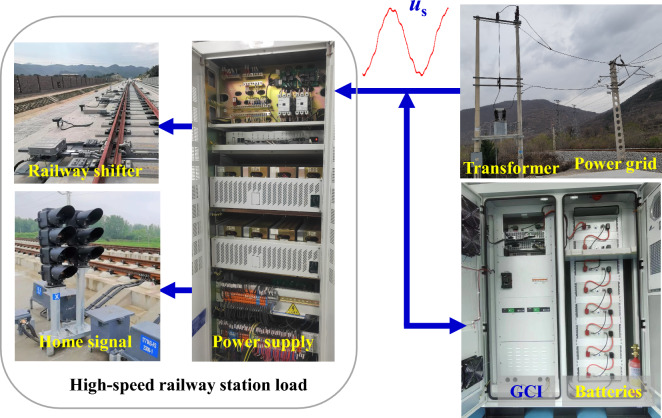
Railway load power supply system.

Figure 14 illustrates the integrated power supply system for the load of a traction substation at a railway station of the National Railway Group, combining the power grid and energy storage. The improved IPT-PLL proposed in this paper was applied to the GCI to accurately capture the phase information of the power grid voltage. The phase-locked loop algorithm was executed using TI's DSP (TMS320F28377) as the central controller. The specific parameters of the experimental setup are outlined in Table 2. The improved IPT-PLL incorporates two park transformations, three LPFs, one PI controller, two resonant controllers, one Lagrange interpolation, and one integral controller. In tests conducted on a floating-point DSP, the execution time was found to be under 19 microseconds, significantly lower than the 100-microsecond switching period of the GCI. Importantly, the operation of the GCI remains unaffected by the improved IPT-PLL. Furthermore, the pointed out PLL program occupies no more than 5 k bits of memory capacity.

**Table 2:** Parameters for the experimental setup.

Nominal conditions	*U* = 311 V, *f*_N_ = 50 Hz
Switching frequency	10 kHz
Parameters of PI controller	*k*_*p*_ = 11.7, *k*_*i*_ = 24,495
Lagrange interpolation polynomial order	N = 3
Parameters of MR controller	*k*_r4_ = 300, *k*_r8_ = 200, *ω*_ch_ = 4 rad/s
Cutoff frequency of the LPF	*ω*_c_ = *ω*_c1_ = 2000π rad/s
The gain of VCO	*k*_*vco*_ = 311

**Figure 15:**
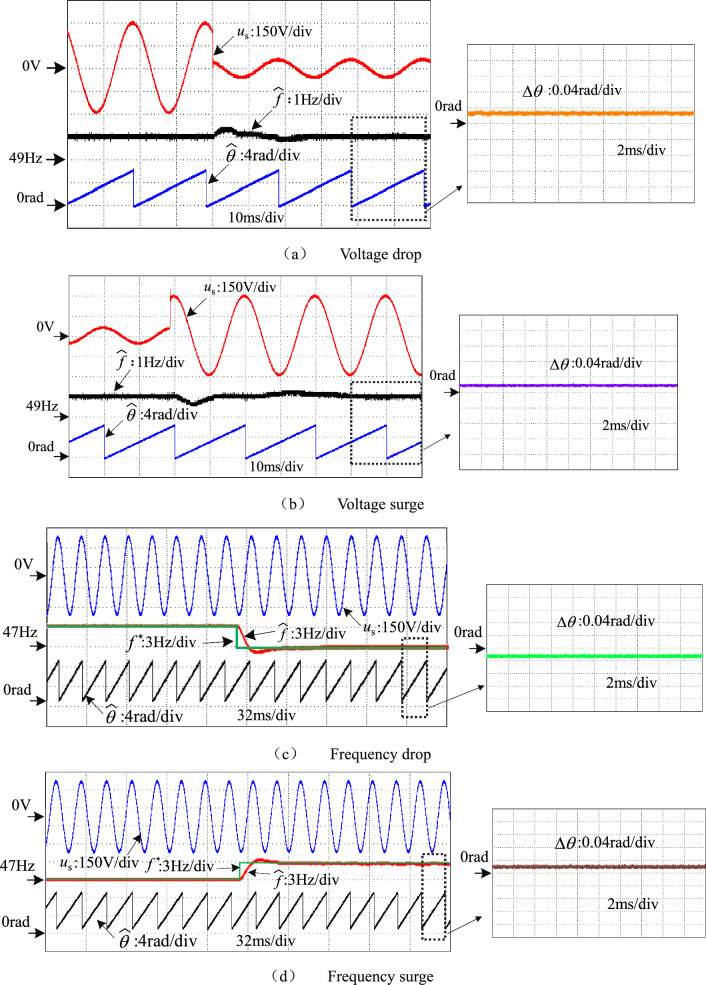
Phase locked waveform of voltage dynamic.

The waveforms of the PLL output frequency $\widehat{f}$, phase $\widehat{\theta }$ and phase error $\vartriangle \theta$ are shown in Fig. 15a,b respectively, when the grid voltage amplitude abruptly drops from 311 to 70 V and abruptly rises from 70 to 311 V. It can be observed that when the grid voltage amplitude undergoes a sudden change, the PLL control system stabilizes within 10 ms. Meanwhile, the steady state phase error of the PLL output does not exceed 0.03 rad. Figure 15c,d show the waveforms of the PLL output frequency $\widehat{f}$, phase $\widehat{\theta }$ and phase error $\vartriangle \theta$ when the grid voltage frequency drops from 50 to 47 Hz and increases from 50 to 53 Hz, respectively. It can be observed that the PLL is able to track the grid frequency accurately within 20 ms and the steady state phase error are no more than 0.02 rad. The improved IPT-PLL scheme enables accurate tracking of the grid frequency.

**Figure 16:**
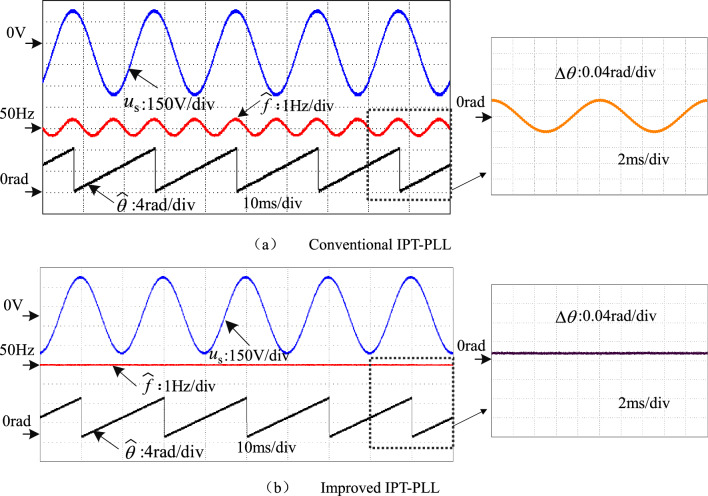
Comparative experiment with DC offset.

Experimental comparisons are conducted to verify the advancement of the improved IPT-PLL over the conventional IPT-PLL. In Fig. 16, a comparative experiment is performed with a grid voltage containing an 8 V DC offset. The output waveforms of the conventional IPT-PLL, shown in Fig. 16a, reveal disturbances at twice the fundamental frequency and phase error due to the DC offset in the grid voltage. However, Fig. 16b displays the experimental waveforms of the improved IPT-PLL. By leveraging the bandpass filter characteristics of *T*_*α*_(s) as described in Eq. (5), the proposed method effectively suppresses the DC component in the grid voltage, thereby eliminating the impact of DC offset on the output frequency of the PLL. Moreover, the phase error remains stable, without any pulsation, and stays below 0.03 radians.

**Figure 17:**
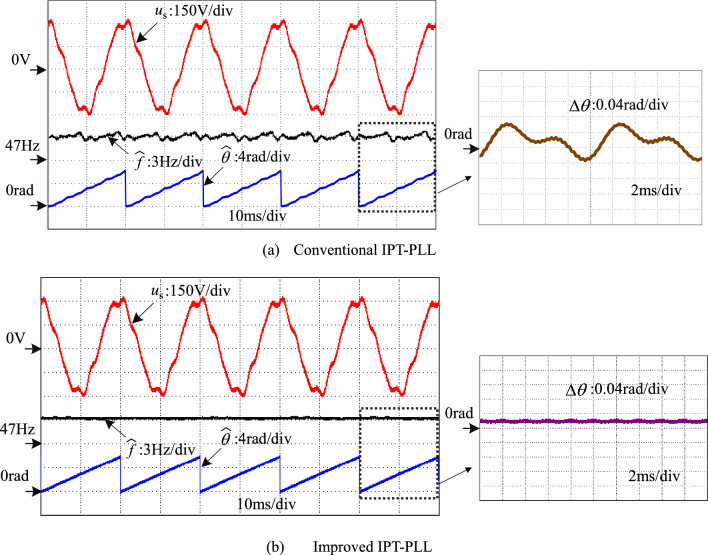
Comparative experiment with low order harmonics.

A comparative experiment in Fig. 17 examines the grid voltage with low-order harmonics, focusing on harmonics ranging from the 3rd to 9th orders. The 3rd harmonic content is at 3%, the 5th harmonic content at 7.5%, the 7th harmonic content at 5.0%, and the 9th harmonic content at 2.0%. In Fig. 17a, the experimental waveform of the conventional IPT-PLL is depicted. It is observed that the output frequency and phase error of the conventional PLL exhibit multiple frequency oscillation components like the 2nd, 4th, and 6th harmonics, impacting the PLL's performance. On the other hand, Fig. 17b showcases the experimental waveform of the improved IPT-PLL. By incorporating multi-harmonic resonant controllers, the PLL's output frequency and phase error display minimal low-order harmonic fluctuations, effectively mitigating the impact of low-order harmonics in the complex power grid voltage on the PLL.

Compared to the conventional IPT-PLL, the improved version not only addresses DC offset and harmonic issues in complex single phase power grid voltage but also mitigates problems arising from voltage frequency variations. The experimental results suggest that the improved IPT-PLL achieves a control accuracy of less than 1% and exhibits commendable dynamic performance.

## Conclusion

This paper presents an improved IPT-PLL technique designed for single-phase GCIs connected to complex single phase power grids. The primary objective of this improvement is to accurately capture fundamental voltage phase and frequency information under conditions with DC offset, low-order harmonics, and fluctuating frequency, thereby facilitating seamless grid connection of inverters.

This study examines the limitations of the traditional IPT-PLL, such as inadequate frequency adaptability, susceptibility to DC offset and low-order harmonics. The improved IPT-PLL incorporates the reverse Park transformation output of the IPT as the reference grid voltage, introducing a T/4 delay to create orthogonal components and implementing a PI + MR controller. Furthermore, a fractional delay approximation of T/4 delay is achieved using Lagrange interpolation polynomial to enhance the frequency adaptability of the phase-locked loop. The design strategies for the PI and MR controllers in the improved IPT-PLL are discussed, followed by a thorough analysis of the system's performance to evaluate its effectiveness. Experimental validation of the proposed technique and design approaches is conducted at a railway station traction substation operated by a national railway group. Results from experiments in complex grid environments show that the fundamental phase of the grid voltage has an error of no more than 1%, with a dynamic time within 10ms. The proposed improved IPT-PLL method shows excellent dynamic and steady-state performance, along with a robust adaptability to complex power grid conditions. In comparison to the conventional IPT-PLL, the improved IPT-PLL exhibits notable advancements (Supplementary Information file 1).

### Supplementary Information

Supplementary Tables.

## Data Availability

All data generated or analyzed during this study are included in this published article [and its supplementary information files]. The datasets used and/or analyzed during the current study are available from the corresponding author on reasonable request.
