# Supplementary material for: An improved IPT-PLL technology for single-phase grid-connected inverters in complex power grid conditions
Source: Sci Rep. 2024 May 28;14:12169. doi: 10.1038/s41598-024-62702-y (PMC11133470; doi:10.1038/s41598-024-62702-y)
Supplement: Supplementary file 1 — Supplementary Tables. [file 41598_2024_62702_MOESM1_ESM.docx]

Appendix A

Table I.  Lagrange interpolation polynomial coefficients for fractional-order delay elements. (N=1,2,3,4）

| N | *d*(0) | *d*(1) | *d*(2) | *d*(3) | *d*(4) |
| --- | --- | --- | --- | --- | --- |
| N=1 | 1-F | F |  |  |  |
| N=2 | (F-1)(F-2)/2 | -F(F-2) | F(F-1)/2 |  |  |
| N=3 | -(F-1)(F-2)(F-3)/6 | F (F-2)(F-3)/2 | -F(F-1) (F-3)/2 | F(F-1)(F-2)/6 |  |
| N=4 | (F-1)(F-2)(F-3)(F-4)/24 | -F(F-2)(F-3)(F-4)/6 | F(F-1)(F-3)(F-4)/4 | -F(F-1)(F-2)(F-4)/6 | F(F-1)(F-2)(F-3)/24 |

Table II. Parameters for the Experimental Setup

| Nominal conditions | *U*=311V, *f*_N_=50Hz |
| --- | --- |
| Switching frequency | 10kHz |
| Parameters of PI controller | *k_p_*=12.3, *k_i_*=19400 |
| Lagrange interpolation polynomial order | N=3 |
| Parameters of MR controller | *k*_r4_ = 300, *k*_r8_ = 200, *ω*_ch_ = 4 rad/s |
| Cutoff frequency of the LPF | *ω*_c_=*ω*_c1_=2000π rad/s |
| The gain of VCO | *k_vco_*=311 |
